# Biomass-Derived Activated Carbon for Congo Red Dye Adsorption: Machine-Learning-Based Prediction and Comparative Evaluation

**DOI:** 10.1021/acsomega.6c02488

**Published:** 2026-05-20

**Authors:** Sujesh Sudarsan, Ramesh Vinayagam, Raja Selvaraj

**Affiliations:** Manipal Institute of Technology, 76793 Manipal Academy of Higher Education, Manipal, Karnataka 576104, India

## Abstract

Congo Red (CR) is a persistent carcinogenic azo dye frequently detected in industrial effluents, requiring reliable tools for predicting its removal performance under variable operating conditions. In this work, machine learning models were used to predict the adsorption behavior of activated carbon derived from *Spathodea campanulata* flowers (SCAC) for the removal of CR dye. A data set of 180 batch experiments (pH: 5–10, dosage: 0.2–1.0 g/L, initial CR: 20–60 mg/L, contact time: 0–180 min, temperature: 293–323 K) was used to construct and evaluate a set of four data-driven modeling approaches, namely support vector machine (SVM), adaptive neuro-fuzzy inference system (ANFIS), artificial neural network (ANN), and multiple linear regression (MLR). As a linear model, the interaction-type MLR model achieved a low R^2^ value (0.8667), indicating only partial capability to represent the nonlinear adsorption behavior. ANFIS showed the strongest overall fit among the tested models (R^2^ = 0.9722), closely followed by ANN (R^2^ = 0.9710). The medium-Gaussian SVM also showed strong predictive ability (R^2^ = 0.9597). Global sensitivity analysis using the optimized ANFIS model ranked the variables as contact time > initial concentration > pH > temperature > dosage. The results demonstrate that ANFIS offers a robust framework for accurately modeling and optimizing CR removal from wastewater by biomass-derived activated carbon.

## Graphical abstract

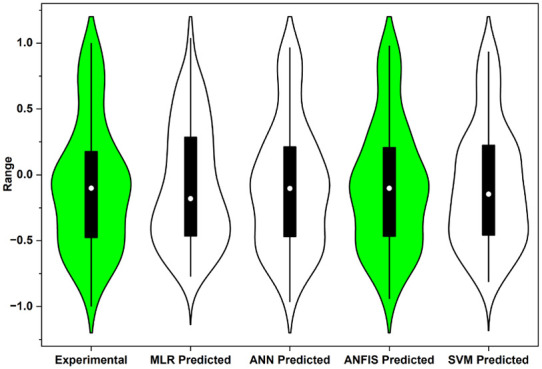

## 1 Introduction

Synthetic dyes are integral to contemporary manufacturing, but these compounds continue to act as significant contributors to persistent water pollution.^1^ Large volumes of colored effluents are discharged from textile, leather, paper, food, and pharmaceutical industries, and many of the dyes possess complex aromatic structures that resist biodegradation.^2^ Once released into aquatic environments, these compounds reduce light penetration, suppress photosynthesis, and may form toxic or mutagenic intermediates, posing serious risks to ecosystems and human health.^3^ Congo Red (CR), an anionic azo dye widely used for cotton and paper dyeing, is particularly problematic because of its carcinogenic and mutagenic potential and its tendency to generate hazardous aromatic amines upon degradation.^4^ Efficient and reliable elimination of CR from wastewater is therefore crucial for responsible water management.

A range of physicochemical and biological processes have been explored for dye treatment, including coagulation–flocculation, advanced oxidation, membrane filtration, and biological treatment. However, these routes are often constrained by high operating costs, incomplete mineralization, strict pH or redox requirements, and the generation of secondary sludge.^5^ By comparison, adsorption stands out as a simple, flexible, and effective alternative, offering high removal efficiency, relatively low capital cost, and easy integration into existing treatment systems. In this context, biomass-derived carbonaceous adsorbents, including biochar (BC)^6^ and activated carbon (AC),^7^ have attracted considerable attention because their adsorption behavior is strongly governed by physicochemical characteristics such as surface morphology, pore structure, mineral composition, and surface functional groups. Among them, AC remains the benchmark adsorbent owing to its high surface area, hierarchical porosity, and rich surface chemistry, which together support multiple interaction mechanisms with dye molecules.^8^ In recent years, there has been growing interest in producing AC from low-cost, renewable biomass residues for targeted dye removal; for example, walnut shell–derived AC for reactive blue 19 and reactive red 195,^9^ bagasse-based AC for methylene blue,^10^ durian seed AC for reactive dyes,^11^ and date palm–derived AC for malachite green (MG).^12^ These biomass-derived carbons lower production costs, reduce reliance on nonrenewable precursors, and promote circular-economy practices by valorizing agricultural and food-processing wastes.

In our earlier work, the underutilized resource, *Spathodea campanulata* flowers, was converted into AC (SCAC) using H_3_PO_4_ activation and moderate-temperature carbonization.^13^ The resulting material exhibited a high specific surface area (986.41 m^2^/g), mesoporous structure, and abundant oxygenated functional groups, which collectively enabled efficient CR uptake. Batch studies indicated that the adsorption kinetics were governed by a pseudo-second-order mechanism, obeyed a Langmuir isotherm with a maximum capacity of 59.27 mg/g, and proceeded via a spontaneous, endothermic mechanism. SCAC also demonstrated good regeneration using methanol and retained significant performance over multiple adsorption–desorption cycles, while tests in natural water matrices confirmed its applicability under realistic conditions.

Although such experimental studies provide valuable mechanistic insight, they are less suited for accurate prediction and process-wide optimization when several operating variables act simultaneously. Classical kinetic and isotherm models typically treat one or two variables at a time and rely on prescribed functional forms, which limit their ability to capture coupled, nonlinear interactions among pH, dosage, contact time, initial dye concentration, adsorbent loading, and temperature. For design and scale-up, however, reliable prediction of adsorption capacity across a wide operating window is crucial. This has motivated the use of data-driven approaches that can learn complex patterns directly from experimental data sets without assuming a specific mathematical structure.

Multiple linear regression (MLR) is frequently adopted as a baseline modeling tool because of its simplicity and interpretability. It relates the response to a linear combination of input variables and can provide a first indication of factor influence.^14^ Nevertheless, its inherent linearity makes it poorly suited to systems where adsorption is governed by film diffusion, intraparticle transport, and multiple surface interactions, all of which are strongly nonlinear. In contrast, modern machine learning (ML) techniques, such as artificial neural networks (ANN),^15^ adaptive neuro-fuzzy inference systems (ANFIS),^16^ and support vector machines (SVM),^17^ are expressly designed to learn nonlinear, high-dimensional relationships and have therefore gained traction in adsorption modeling.

Recent studies show a growing shift toward ML-based adsorption prediction. Nguyen et al. developed an ANN model for CR uptake on microwave-synthesized akaganeite nanoparticles,^18^ while Ahmad et al. applied ANN to describe sunset yellow adsorption onto neodymium-modified ordered mesoporous carbon.^19^ In parallel, ANFIS has been shown to capture CR removal on chemically modified clays with good predictive accuracy and interpretability.^20^ SVM-based regression has likewise been applied to methyl orange adsorption on CTAB-functionalized graphene oxide (CTAB@GO),^21^ and to the simultaneous removal of bisphenol A and Acid Red 1 dyes using a polyethylenimine/graphene oxide/layered triple hydroxide nanocomposite, where it delivered high predictive accuracy even with relatively small data sets.^22^ Together, these contributions highlight the promise of ML for adsorption modeling, but they typically examine a single algorithm applied to nonbiomass-based sorbents. To date, a direct comparative study of ANN, MLR, ANFIS, and SVM approaches for modeling CR removal using a biomass-based adsorbent remains unreported. Moreover, the relative influence of operating variables within such models has seldom been quantified using global sensitivity analysis. Recently, our group implemented ANN and ANFIS frameworks to model MG uptake using superparamagnetic AC derived from *S. campanulata* flowers.^23^

The present study addresses these gaps by developing and comparing four predictive models, such as ANN, SVM, ANFIS, and MLR, for CR uptake onto SCAC, using the comprehensive experimental data set from our earlier work. The models take solution pH, adsorbent dosage, initial CR concentration, contact time, and temperature as inputs and predict the equilibrium adsorption capacity Q_e_. Because the selected algorithms follow different training structures, model performance was evaluated using model specific validation workflows. MLR and SVM were assessed using 5-fold cross validation, whereas ANN and ANFIS were evaluated using independent training, validation or checking, and testing subsets. The prediction quality was then interpreted using standard statistical indicators, including coefficient of determination, R^2^, mean squared error (MSE), root mean squared error (RMSE), and mean absolute error (MAE), together with subset specific generalization behavior. In addition, an ANFIS-based global sensitivity analysis is carried out to rank the influence of the operating variables and to relate the data-driven findings to known adsorption mechanisms. By integrating sustainable adsorbent development with advanced ML modeling, this work aims to provide a reliable predictive framework for the design and optimization of CR removal processes using biomass-derived AC.

## 2 Methodology

### 2.1 Data Set Compilation and Preprocessing

The data sets used to develop the MLR, ANFIS, SVM, and ANN frameworks were generated from our earlier published data set on CR removal using SCAC.^13^ Five operating variables were considered as model inputs: solution pH, initial CR concentration (C, mg/L), adsorbent dosage (D, g/L), temperature (T, K), and contact time (t, min). The equilibrium adsorption capacity, Q_e_ (mg/g), was treated as the sole predicted output. In total, 180 batch experiments were compiled, covering the ranges: pH = 5–10, D = 0.2–1.0 g/L, C = 20–60 mg/L, t = 0–180 min, and T = 293–323 K, yielding adequate diversity to support rigorous model training and testing. The present data set size is also larger than several earlier ANN and ANFIS based adsorption modeling studies, such as Kumari et al., who used 30 experimental runs for methylene blue adsorption modeling,^24^ and Esfandyari et al., who used 41 data sets for Ni­(II) removal and 43 data sets for Pb­(II) removal.^25^ Before model development, all input and output variables were rescaled to the interval ±1 using min–max normalization to avoid bias arising from differences in units and magnitude. All subsequent modeling and simulations were carried out in MATLAB R2020b (MathWorks, USA).

### 2.2 MLR Modeling

MLR expresses one response as a linear combination of several predictors and is favored for its simplicity, interpretability, and computational efficiency.^14^ The estimated coefficients indicate the direction and magnitude of each factor’s effect on Q_e_. In this work, three MLR variants were implemented within the MATLAB Regression Learner Toolbox: standard linear, interaction linear, and robust linear. Model calibration and evaluation were carried out using 5-fold cross-validation. The data set was divided into five subsets; in each round, four parts were used for training, and the held-out part served for validation. This strategy reduces overfitting and provides more reliable estimates of predictive performance for new, unseen data.^26^

### 2.3 ANN Architecture

ANNs are data-driven function approximators that draw on principles of biological information processing. They comprise layered collections of interconnected units called neurons, linked by trainable weights and activation functions, allowing the network to learn complex nonlinear mappings from input variables to target outputs.^27^ In the present work, an ANN was developed to predict Q_e_ from the five operating parameters (pH, D, C, t, and T). The neural network was implemented in MATLAB 2020b (MathWorks, USA) through the Neural Net Fitting interface of the Deep Learning Toolbox (Figure 1a). The model architecture comprised an input layer with five neurons, a single hidden layer, and an output layer with one neuron representing Q_e_. The number of neurons in the hidden layer was selected by trial and error, by testing configurations with 2 to 14 neurons. Network training was carried out using the Levenberg–Marquardt backpropagation algorithm, which was employed for its efficiency in convergence and robust learning stability.^28^ A tangent sigmoid (*tansig*) transfer function was assigned to the hidden layer, while a linear (*purelin*) transfer function was used in the output layer to preserve the continuous scale of Q_e_. A total of 180 experimental data points were randomly divided into training, validation, and testing groups in a 70–15–15 allocation.^29^ During training, the validation subset was used to monitor generalization and to stop learning before overfitting occurred, whereas the test subset was kept aside until the final stage and provided an independent estimate of predictive accuracy.

**Figure 1:**
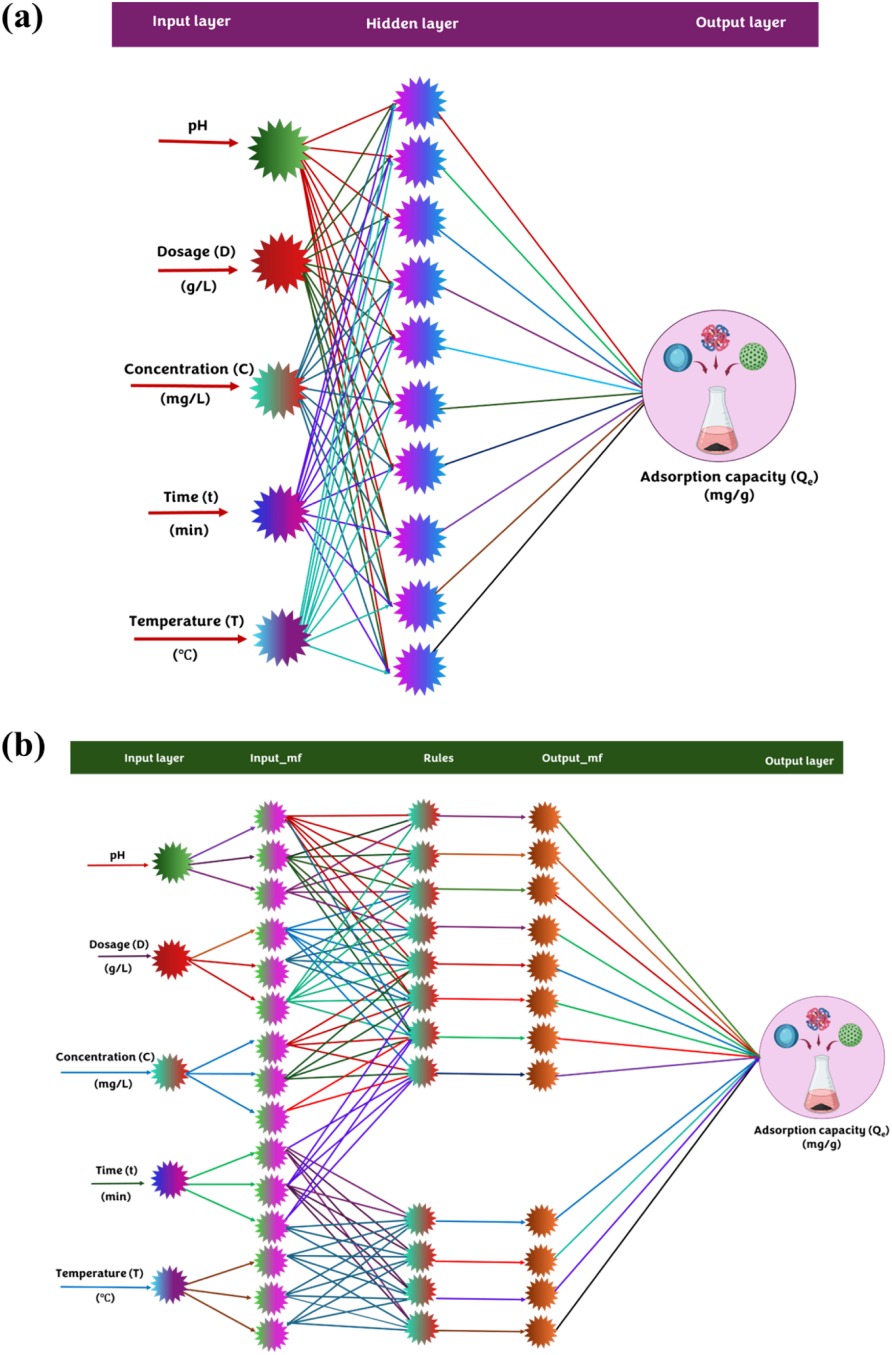
Architectures of the ML models used for predicting CR adsorption on SCAC: (a) artificial neural network (ANN) and (b) adaptive neuro-fuzzy inference system (ANFIS).

### 2.4 ANFIS Configuration

ANFIS combines the adaptive learning mechanisms of neural networks with the inference structure of fuzzy logic systems, enabling the modeling of nonlinear responses and uncertainty in process variables.^30^ In this framework, fuzzy membership functions (MFs) define the input space, while data-driven training adjusts their parameters, providing both predictive accuracy and interpretability for complex adsorption systems.

In the present study, ANFIS was employed to predict Q_e_ from the same five input variables used in the ANN model. The adopted architecture comprised five layers:^31^ input, fuzzification, rule, defuzzification, and output (Figure 1b). The predictor variables were first passed through the input layer and subsequently mapped into fuzzy representations within the fuzzification layer using first-order Sugeno-type MFs.^32^ Within the rule layer, these fuzzy inputs were combined using a set of “*if–then*” rules, after which the defuzzification layer converted the aggregated fuzzy outputs into crisp values. The output layer then combined these contributions into a single predicted value of Q_e_. The ANFIS model was constructed in MATLAB through the *anfisedit* interface, and parameter learning was performed with the standard hybrid learning algorithm.^24^

The same data set used for ANN was employed here and segregated into training (70%), checking (15%), and testing (15%) subsets. The training subset was used to calibrate the parameters of the fuzzy inference system, while the checking subset monitored generalization and assisted in selecting suitable MF configurations. To improve predictive performance, both the type and number of MFs were systematically evaluated. The MF types examined included *Gaussian (gaussmf), pi-shaped (pimf), generalized bell (gbellmf), hybrid Gaussian (gauss2mf), sigmoidal (dsigmf), triangular (trimf), and trapezoidal (trapmf)*. For each input, configurations with two to five membership functions were examined (2–2–2–2–2 to 5–5–5–5–5). To minimize overfitting and maintain statistical rigor, the maximum training iterations were limited to 10.

### 2.5 SVM Modeling

Support vector machine (SVM) is a supervised learning framework that relies on kernel-induced feature mappings to transform the original input space into a higher-dimensional representation, where the dependencies between variables can be expressed through a linear function.^33^ In regression mode, SVM determines an optimal hyperplane that balances model complexity and prediction error, enabling it to capture nonlinear dependencies between inputs and the response. Its predictive behavior, however, is largely dictated by the choice of kernel mapping and the tuning of regularization hyperparameters that regulate the margin width and the permissible error threshold.^22^

A range of kernel options was examined, encompassing linear kernels, polynomial kernels of second and third order, and Gaussian radial basis kernels configured with fine, medium, and coarse types. SVM model construction and parameter tuning were performed in MATLAB through the Regression Learner toolbox. As with the MLR models, 5-fold cross-validation was adopted to assess generalization performance, with each fold being used once as the validation subset, while the remaining folds served as the training data. The averaged statistical metrics over all folds were then taken as representative indicators of the predictive quality of each SVM configuration.

### 2.6 Sensitivity Assessment

Following the training and validation of the optimized ANFIS structure (Sugeno type, 3–3–3–3–3 structure with *trimf*), a global sensitivity analysis was carried out to quantify the influence of each operating parameter on Q_e_. In this procedure, one input variable (pH, D, C, t, or T) was systematically altered over its operational window, with the other inputs held at their mean levels. The corresponding changes in the ANFIS predicted Q_e_ were used to construct partial dependence plots for each parameter. The Q_e_ responses were then normalized to obtain the relative contribution of each variable, which was finally used to rank their influence on CR adsorption.

### 2.7 Comparative Evaluation of the Models

For the CR–SCAC adsorption system, the performance of all predictive models was assessed using four standard statistical criteria: R^2^, MSE, RMSE, and MAE, expressed in eqs 1–4:

1 $$R^{2}=1-\frac{\overset{n}{\underset{i=1}{\sum }}{[Q_{ei,\mathrm{experimental}}-Q_{ei,predicted}]}^{2}}{\overset{n}{\underset{i=1}{\sum }}{[Q_{ei,\mathrm{experimental}}-Q_{ei,average}]}^{2}}$$

2 $$MSE=\frac{1}{n}\overset{n}{\underset{i=1}{\sum }}{[Q_{ei,\mathrm{experimental}}-Q_{ei,predicted}]}^{2}$$

3 $$RMSE=\sqrt{\frac{1}{n}\overset{n}{\underset{i=1}{\sum }}{[Q_{ei,\mathrm{experimental}}-Q_{ei,predicted}]}^{2}}$$

4 $$MAE=\frac{1}{n}\overset{n}{\underset{i=1}{\sum }}\left|Q_{ei,experimental}-Q_{ei,predicted}\right|$$

Here, Q_e_ represents the adsorption capacity (mg/g), and n is the number of experimental data points. R^2^ measures how well the predicted values reproduce the variance of the experimental data, whereas MSE, RMSE, and MAE quantify the magnitude of prediction errors. In the present study, these statistical indices were interpreted together with subset specific evaluation. For the ANFIS model, the overall values were treated only as pooled summary indicators across the available data subsets, whereas the assessment of generalization was based primarily on the training, checking, and testing results, with particular emphasis on the held-out testing error. Accordingly, the most suitable model was identified not only from lower overall error and higher R^2^, but also from its ability to maintain consistent predictive performance on unseen data.

## 3 Results and Discussion

### 3.1 Pearson Correlation Analysis

The correlation heatmap shown in Figure 2 was used to understand how the experimental variables of the SCAC adsorption system, namely pH, D, C, t, and T, are linearly related to the equilibrium uptake Q_e_. Pearson’s correlation produces a coefficient between −1 and +1. Values that are nearer to +1 indicate a strong direct relation, values that are nearer to −1 indicate an inverse relation, and values around zero indicate that the two quantities do not vary together in a linear manner.^34^

**Figure 2:**
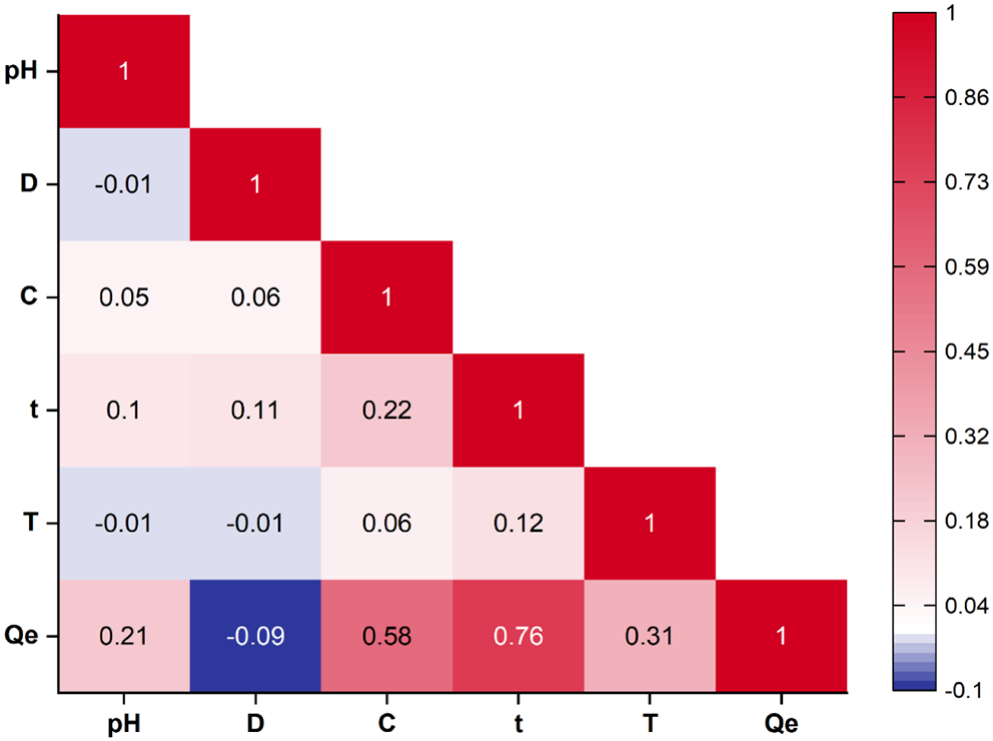
Pearson correlation matrix showing relationships among input variables (pH, D, C, t, T) and the response variable (Q_e_) for the CR–SCAC system.

The matrix clearly shows that t is the most influential variable. Its correlation with Q_e_ is 0.76, which means that longer interaction of the CR dye with the SCAC surface consistently results in higher adsorption. This agrees with the batch adsorption behavior already reported for SCAC, where dye removal increased steadily with time, as external film diffusion occurred first, followed by the penetration of dye molecules into the mesoporous network until the internal sites became occupied.^13^ A comparable observation was made by Thabet et al., who reported that contact time exhibited the highest positive correlation (r = 0.53) with methylene blue removal efficiency, confirming that longer contact durations substantially enhance diffusion and surface-site utilization.^35^ The second strongest relation is between C and Q_e_, with a coefficient of 0.58. When the feed contains more dye, the driving force for mass transfer is higher, collisions with active sites are more frequent, and a larger amount of dye is finally adhered to the carbon surface.^36^ T has a moderate but still positive correlation with Q_e_, with a value of 0.31, which supports the earlier thermodynamic evidence for an endothermic process. A higher temperature supplies additional kinetic energy to the dye molecules, reduces diffusional resistance, and promotes their movement toward the interior adsorption sites.^37^

In contrast, pH and D show only weak links with Q_e_. The correlation between pH and Q_e_ is 0.21, which means that within the pH window used in this study, the surface charge of SCAC and the ionization state of CR change only slightly, so electrostatic attraction improves only to a limited extent.^38^ D even shows a small negative relation with Q_e_, with a value of −0.09. This is a common observation in batch adsorption since Q_e_ is expressed per unit mass of adsorbent. When more carbon is added while the dye concentration is kept the same, the available sites are not fully utilized, and slight particle aggregation may occur, both of which reduce the amount adsorbed per gram of adsorbent. Slight but noticeable interrelationships were also found among the input variables. The correlation between C and t was 0.22, while that between t and T was 0.12. These modest positive associations suggest that an increase in dye concentration or temperature enhances diffusion rates and adsorption kinetics, thereby influencing the time required to reach equilibrium. Importantly, since none of these pairwise correlations exceed 0.8, serious multicollinearity can be ruled out, confirming that the selected variables are largely independent and suitable for subsequent multivariate and ML modeling.^39^

### 3.2 MLR Modeling

MLR served as the preliminary modeling framework to characterize the relationship between the process variables and Q_e_. Among the tested configurations, the interaction linear type demonstrated superior predictive capability (Table 1), yielding an R^2^ of 0.8667 accompanied by minimal prediction errors (RMSE = 0.1773, MSE = 0.0314, MAE = 0.1385). This improvement indicates that including interaction terms between variables provided a more realistic representation of the system compared to the simple linear (R^2^ = 0.8400) and robust linear (R^2^ = 0.8000) models. The result suggests that adsorption behavior is not governed by individual parameters alone but also by their combined effects, such as the interplay between concentration and time or between dose and pH, which significantly influences the overall adsorption efficiency.

**Table 1:** Statistical Performance of Different MLR Models for Predicting CR Adsorption on SCAC

	Linear	Interactions Linear	Robust Linear
R^2^	0.8400	0.8667	0.8000
MSE	0.0376	0.0314	0.0475
RMSE	0.1940	0.1773	0.2179
MAE	0.1477	0.1385	0.1456

The scatter plot between experimental and predicted Q_e_ values (Figure S1) supports this observation, as most data points lie close to the one-to-one parity line, indicating a strong concordance between the measured and model-estimated values. However, a gradual deviation from the ideal line is observed at higher Q_e_ values. This deviation arises because, under high initial dye concentrations and prolonged contact times, the SCAC surface becomes progressively saturated, and intraparticle diffusion as well as multiple concurrent adsorption mechanisms begin to dominate.^13^ These mechanisms are inherently nonlinear and therefore cannot be fully captured by a linear regression approach. As a result, the MLR model performs reliably within the low-to-moderate adsorption range, where the relationship between variables and response remains almost linear, but loses accuracy at higher adsorption capacities where nonlinear effects prevail. Comparable observations were made for 2,4-Dichlorophenoxyacetic acid (2,4-D) adsorption on rice husk biochar, where classical MLR could describe the data only to some extent (R^2^ = 0.9453), but was clearly outperformed by a nonlinear ML algorithm, such as random forest, mainly because the adsorption variables and uptake showed a nonlinear relationship.^40^ Hence, for more precise and reliable predictions, advanced nonlinear ML models such as ANN, ANFIS, and SVM would be more suitable to capture the intricate mass-transfer and surface-interaction dynamics of the system.

### 3.3 ANN Architecture

A feed-forward back-propagation network with one hidden layer was used, and the number of neurons in this hidden layer was tuned by trial and error. Figure 3a shows that, when the hidden neurons were increased from 2 to 10, the overall correlation coefficient (R) steadily rose while MSE fell, meaning the network was learning the input–output pattern better. The best response was obtained at 10 neurons, where the model recorded the highest R (0.9854) together with the lowest MSE (0.0068). This 5–10–1 structure was therefore chosen as the optimal architecture because it gives the right balance between model flexibility and generalization.

**Figure 3:**
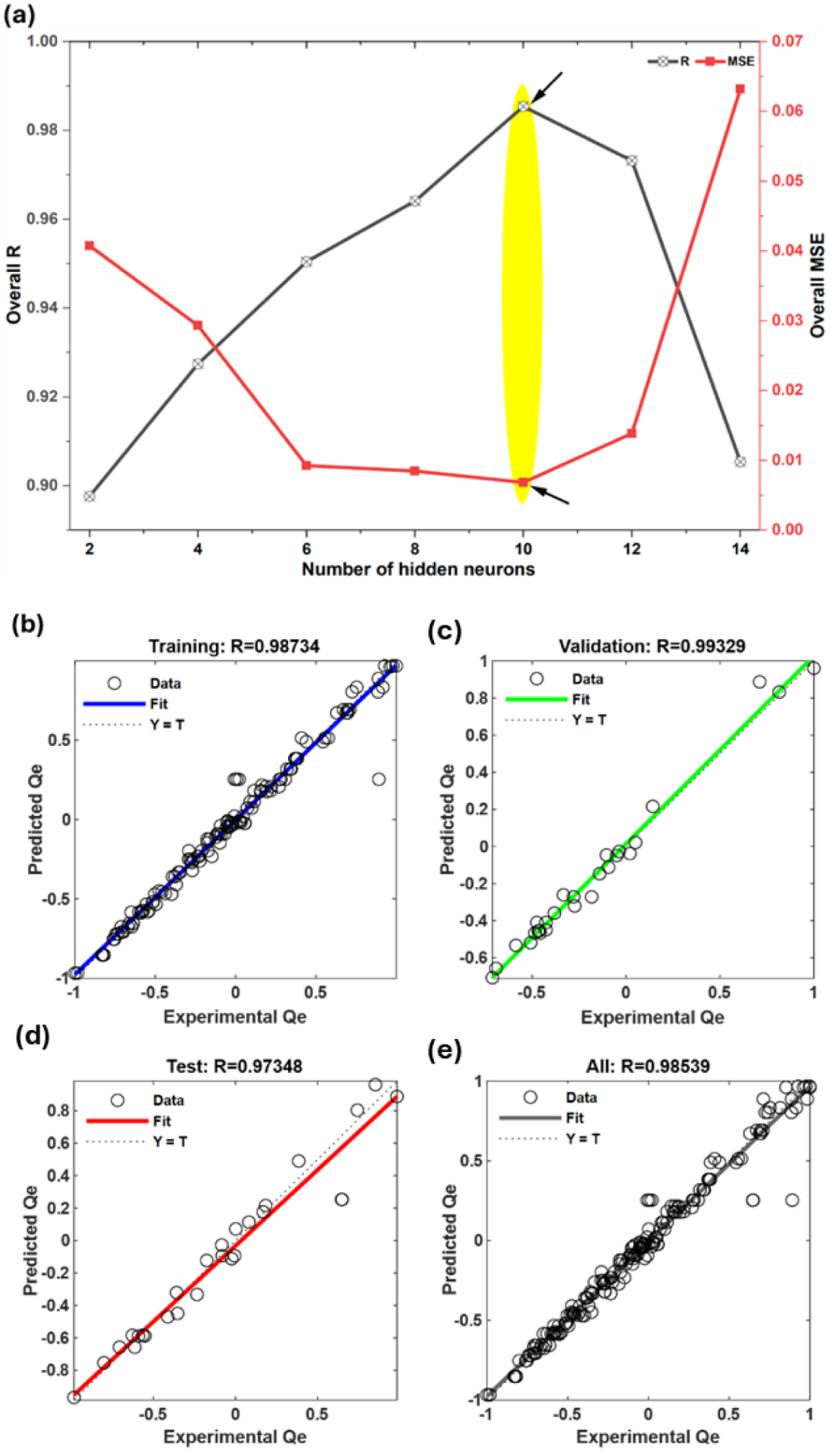
ANN model optimization and validation for CR adsorption on SCAC: (a) effect of hidden neurons on R and MSE; correlation between experimental and predicted Q_e_ for (b) training, (c) validation, (d) testing, and (e) overall data sets.

When the neurons were increased further, the performance dropped; at 14 neurons, R decreased to 0.9054, and MSE increased sharply to 0.0632, indicating the start of overfitting, i.e., the network began to memorize the training data instead of learning the real process trend. Similar optimization trends have been observed in earlier adsorption-based ANN studies. For instance, phenol red dye adsorption on gold-loaded AC exhibited maximum predictive accuracy at 15 hidden neurons, beyond which the model’s performance declined due to overfitting.^41^ In another study, 8 neurons yielded the best configuration for modeling 2,4-D removal using an AC/poly dimethylaminoethyl methacrylate hydrogel,^42^ while Cu­(II) adsorption on sodium-activated Philippine zeolite achieved its optimal learning efficiency with 7 neurons.^27^ These consistent patterns across different adsorption systems confirm that network performance typically improves with increasing neuron number up to an optimal point, after which excessive neurons lead to diminished generalization and higher prediction error.

The regression plots of the selected 5–10–1 model further confirm its prediction quality. Data points for training (Figure 3b), validation (Figure 3c), and testing (Figure 3d) are all closely aligned with the 1:1 line, showing that the model can reproduce experimental Q_e_ values over the entire range of conditions. The network achieved R values of 0.98734 (training), 0.99329 (validation), and 0.97348 (testing), which means that more than 97–99% of the variance in Q_e_ is explained in every phase. Unlike linear models, the ANN could also map the high-uptake region, where surface saturation, intraparticle diffusion, and multiple interaction forces make the relationship strongly nonlinear. Therefore, the optimized ANN provides a compact and accurate soft-computing model for the adsorption. It outperforms the linear regressions by recognizing the combined effect of the five process variables, preserving good generalization without overfitting at 10 hidden neurons, and giving very low prediction error even at high Q_e_ values. This makes the ANN suitable for process prediction and for later use in optimization or decision-support modules.

### 3.4 ANFIS Configuration

#### 3.4.1 Optimization of the Number of Membership Functions

The adsorption data set was modeled using an ANFIS framework, where all five input variables were assigned an equal number of triangular membership functions (*trimf*) for uniformity.^43^ Model performance was evaluated using RMSE for the training, checking, and testing data sets, as summarized in Table S1.

When two membership functions were used for each input variable (2–2–2–2–2), the network exhibited clear underfitting, with training, checking, and testing RMSE values of 0.1087, 0.0706, and 0.5090, respectively. The high testing error indicated that the model capacity was insufficient to capture the complex nonlinear relationship between the process parameters and dye adsorption capacity, necessitating a higher number of membership functions.^24^ Increasing the number of membership functions to three for each input (3–3–3–3–3) markedly improved performance. The training RMSE decreased to 0.0573, while the checking RMSE reached its lowest value of 0.0576, and the testing RMSE dropped substantially to 0.1585. This configuration demonstrated the best balance between accuracy and generalization, effectively learning the underlying adsorption patterns without overfitting. Further increasing the number of membership functions to four (4–4–4–4–4) and five (5–5–5–5–5) produced only marginal gains. Although the training RMSE slightly decreased to 0.0566 and 0.0539, the checking RMSE increased (0.0597 and 0.0617), while the testing RMSE remained nearly unchanged (0.1590 and 0.1583). These results indicate that additional membership functions mainly improved fitting on the training data but did not enhance predictive performance, reflecting the classic bias–variance trade-off.^44^ Notably, an ANFIS model developed for chromium adsorption using green adsorbents achieved its best performance with a 4–4–4 configuration, underscoring that the optimal number of membership functions can vary depending on the data set and adsorption system characteristics.^45^ In the present study, the 3–3–3–3–3 configuration was therefore selected as the most suitable structure because it provided the best compromise between calibration accuracy and generalization, as indicated by the lowest checking RMSE and the most favorable testing performance among the ANFIS configurations examined. Thus, final model selection was guided by the combined training, checking, and testing errors rather than by training fit alone.

#### 3.4.2 Selection of Membership Function Type

With the ANFIS structure fixed at the 3–3–3–3–3 topology, several MF types were evaluated to identify the most suitable representation for the adsorption system (Figure 4) . Among these, the *trimf* provided the most stable and accurate performance across all data sets. The *gauss2mf* configuration produced a slightly lower training error but exhibited a higher testing RMSE, indicating mild overfitting; the additional smoothness of the Gaussian pair did not yield better predictive accuracy. Other MF types (*trapmf, gbellmf, gaussmf, pimf, and dsigmf*) showed comparable training performance but consistently higher checking and testing errors than *trimf*, confirming that they were less effective for this data set. Interestingly, prior research on copper adsorption using biochar reported that *gaussmf* offered the best performance,^46^ highlighting that the optimal MF selection is system-specific and must be determined through data set-driven validation rather than a fixed default choice.

**Figure 4:**
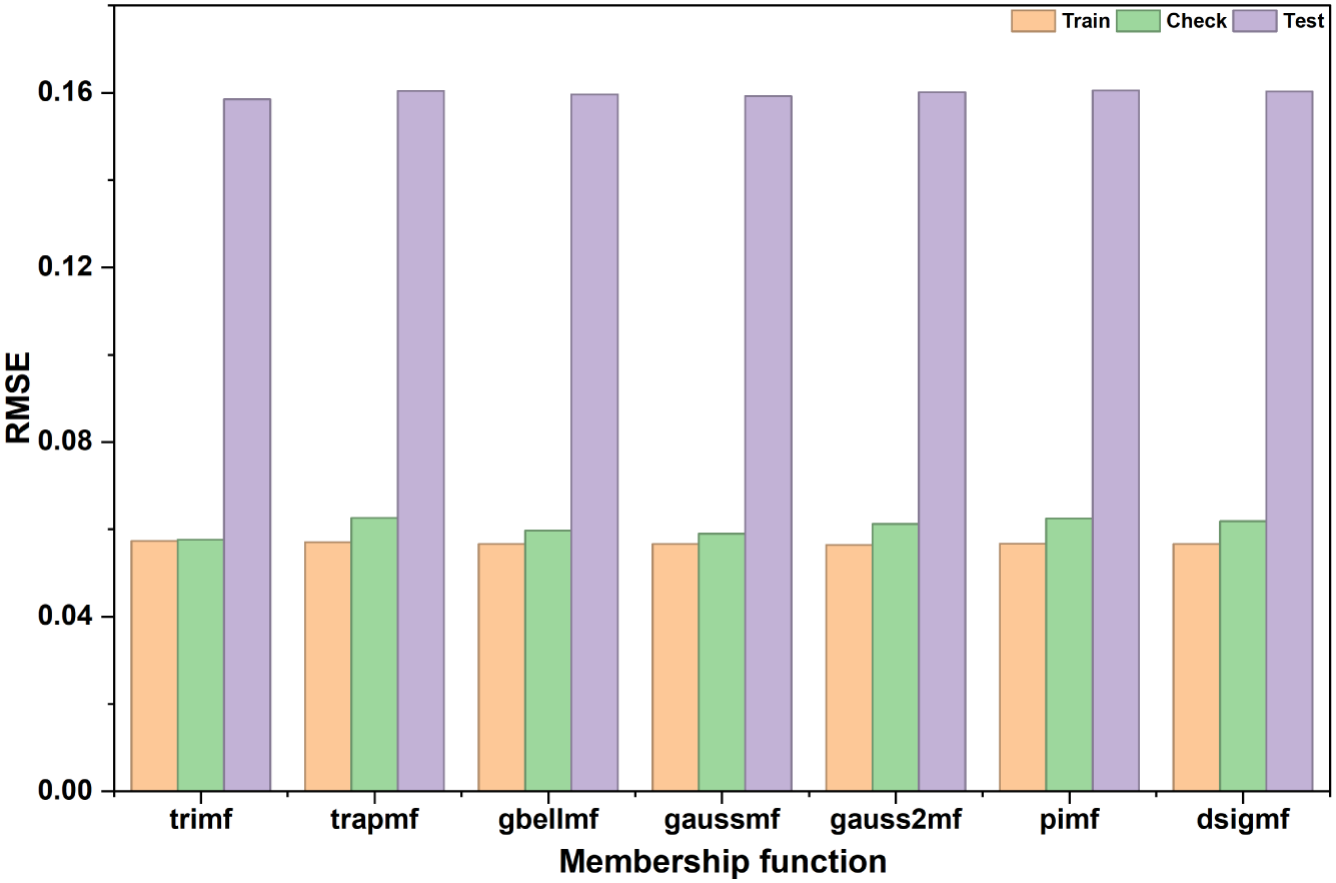
ANFIS (3–3–3–3–3) model performance with different membership functions based on RMSE for training, checking, and testing data sets.

The corresponding statistical indicators of *trimf* for this configuration are presented in Table S2, showing high coefficients of R^2^ (0.9868 for training, 0.9857 for checking, and 0.9722 overall) and low error metrics (RMSE = 0.0573, 0.0576, and 0.1585 for training, checking, and testing, respectively). These results confirm that the *trimf*-based model effectively captured the nonlinear adsorption relationship between operating variables and dye uptake while maintaining excellent generalization.

#### 3.4.3 Performance of the Optimized ANFIS Structure

The performance of the optimized ANFIS configuration is presented in Figure 5, while the key model parameters are summarized in Table 2. As illustrated in the error curve (Figure 5a), both the training and checking RMSE values decreased sharply during the initial epochs and stabilized after the fifth epoch, indicating rapid convergence and stable learning without signs of overfitting or divergence. The minimal gap between training and checking errors indicates stable calibration behavior of the selected structure.^47^ The comparison between predicted and experimental outputs for the training (Figure 5b), checking (Figure 5c), and testing (Figure 5d) data sets showed a close one-to-one correspondence. Although the testing subset showed a higher prediction error than the calibration subsets, the selected ANFIS structure provided the most favorable compromise between fitting accuracy and predictive consistency among the tested configurations. Altogether, the developed ANFIS model effectively represented the nonlinear adsorption behavior of CR onto SCAC while retaining interpretability.

**Table 2:** Key Parameters of the Optimized ANFIS (3–3–3–3–3) Model for Predicting CR Adsorption on SCAC

FIS type	Sugeno
Input MF	*trimf*
Output MF	Linear
Optimization method	Hybrid
Topology	3–3–3–3–3
Number of nodes	524
Number of linear parameters	1458
Number of nonlinear parameters	45
Total number of parameters	1503
Number of training data pairs	126
Number of checking data pairs	27
Number of fuzzy rules	243

**Figure 5:**
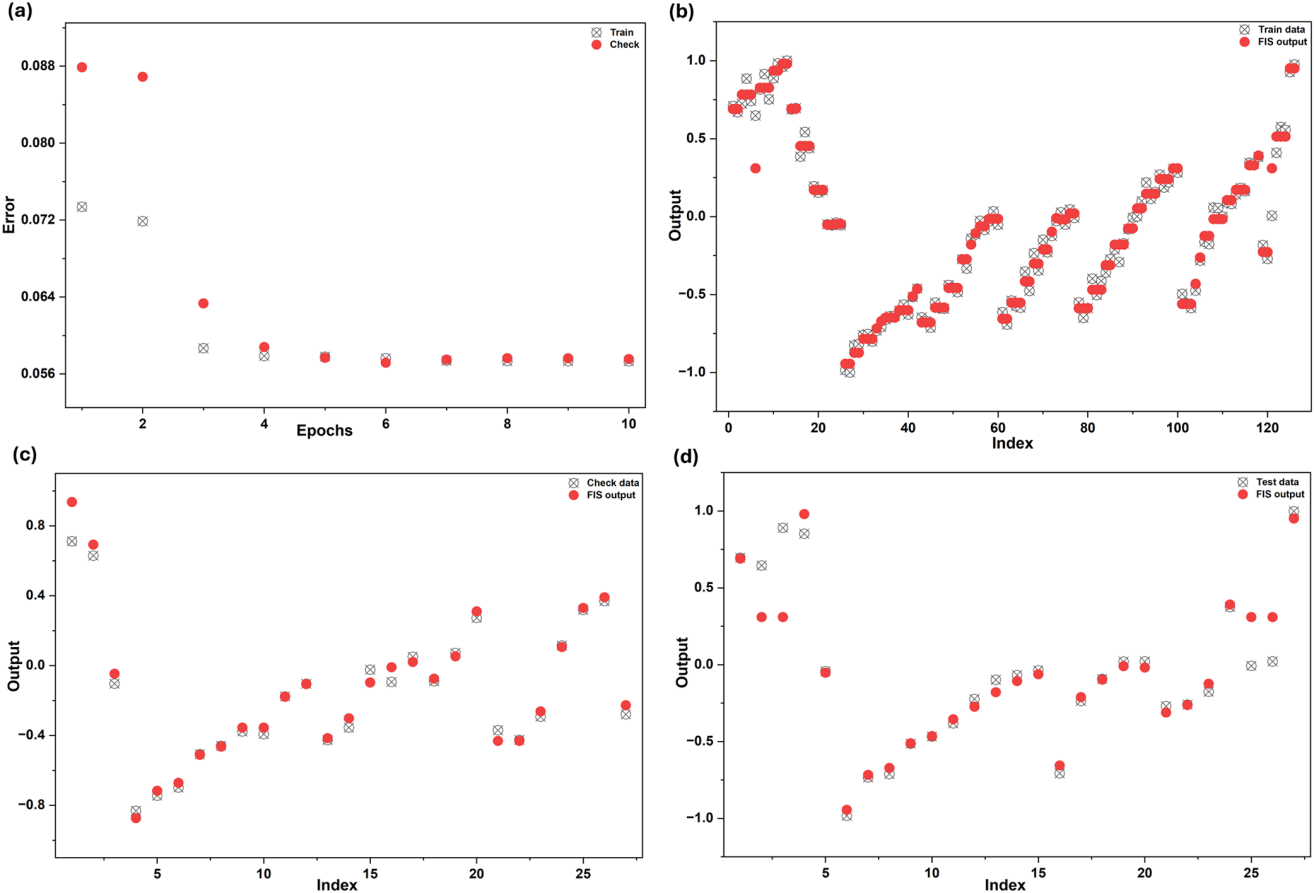
Training, checking, and testing performance of the optimized ANFIS (3–3–3–3–3) model: (a) error convergence with epochs; (b) training; (c) checking; and (d) testing predictions versus experimental outputs.

### 3.5 SVM Modeling

To complement the fuzzy–hybrid approach, the adsorption data set was also modeled using support vector regression (SVR) with different kernel functions. Six kernel types, such as linear, quadratic, cubic, fine Gaussian, medium Gaussian, and coarse Gaussian, were trained on the same input–output pairs, and their statistical indices (R,^2^ MSE, RMSE, and MAE) are compared in Figure 6. The medium Gaussian (radial basis) kernel was clearly the best performer. It yielded the highest coefficient of determination (R^2^ = 0.9597) together with the lowest error values (MSE = 0.0099, RMSE = 0.0995, MAE = 0.0682), corroborating that it captured the nonlinear dependence of CR uptake on operating variables more effectively than the other kernels. The fine Gaussian model, which uses a narrower bandwidth, produced comparable accuracy but with slightly higher RMSE and MAE, suggesting mild sensitivity to noise. Polynomial kernels (quadratic and cubic) improved substantially over the linear model, but still could not match the flexibility of the medium Gaussian. The coarse Gaussian and linear kernels showed the weakest approximation capability, reflecting underfitting of the nonlinear adsorption surface.

**Figure 6:**
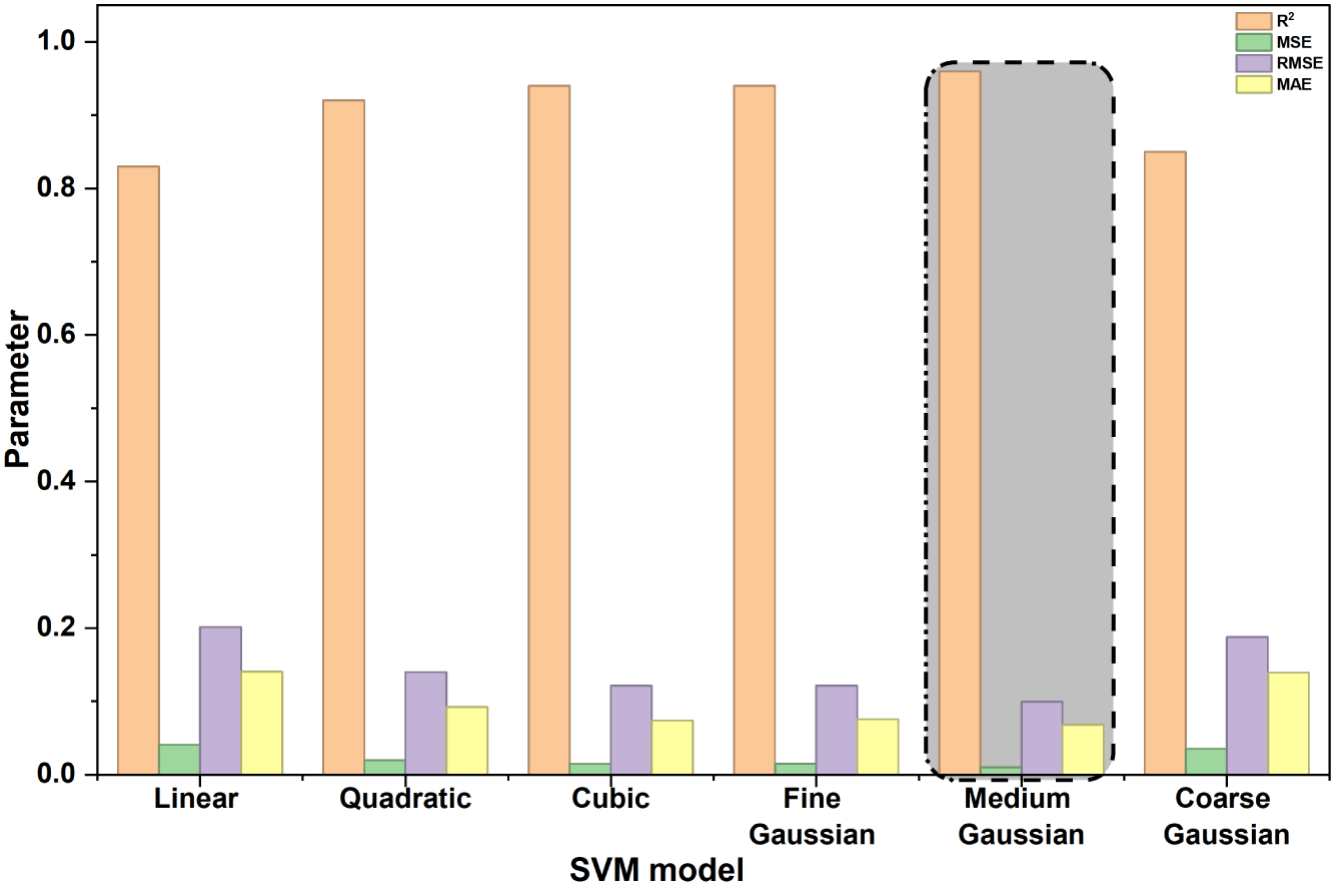
Comparative performance of SVM models with different kernel functions using R^2^, MSE, RMSE, and MAE.

The scatter plot of predicted versus experimental adsorption capacity for the medium Gaussian model (Figure S2) further supports this conclusion. Data points are densely aligned along the 1:1 line with only small deviations at the extremes, confirming that the trained SVR generalized well. Thus, the medium Gaussian kernel can be regarded as the most suitable SVM configuration for this data set. It is worth noting that SVM performance in adsorption problems is strongly data set-dependent; for example, an SVM built for heavy-metal uptake on biochar reported an even higher R^2^ (0.9832),^48^ indicating that the attainable accuracy is governed by the smoothness and scatter of the experimental data.

### 3.6 Sensitivity Analysis of Process Variables

Global sensitivity evaluation using the optimized ANFIS model identified the relative and interactive effects of operating parameters on Q_e_ (Figure 7f). Among all variables, contact time (t) exhibited the strongest influence, with Q_e_ increasing steadily until equilibrium (Figure 7d), underscoring the dominant role of prolonged interaction in promoting complete monolayer formation, as reflected by the superior fit of the Langmuir isotherm in batch experiments and by enhanced intraparticle diffusion.^49^ Initial dye concentration (C) ranked second (Figure 7c), as higher concentration gradients enhanced the mass-transfer driving force and increased Q_e_ before a slight decline at very high levels due to site saturation.^50^ pH followed, showing a gradual decrease in Q_e_ with increasing values (Figure 7a), indicating that acidic conditions strengthen electrostatic attraction between CR molecules and positively charged AC surfaces.^13^ Temperature (T) occupied the next position (Figure 7e), reflecting the endothermic nature of adsorption, where higher thermal energy accelerates molecular motion and facilitates diffusion.^9^ Adsorbent dosage (D) contributed the least (Figure 7b), giving only minor improvements that diminished at higher loadings because of particle aggregation and diffusion resistance.^51^ The overall order of influence obtained from sensitivity analysis was t > C > pH > T > D. These findings show that CR dye uptake is mainly controlled by kinetic effects, mass transfer, and surface charge, while temperature and adsorbent dosage have smaller supporting roles, giving a clear basis for process optimization and scale-up.

**Figure 7:**
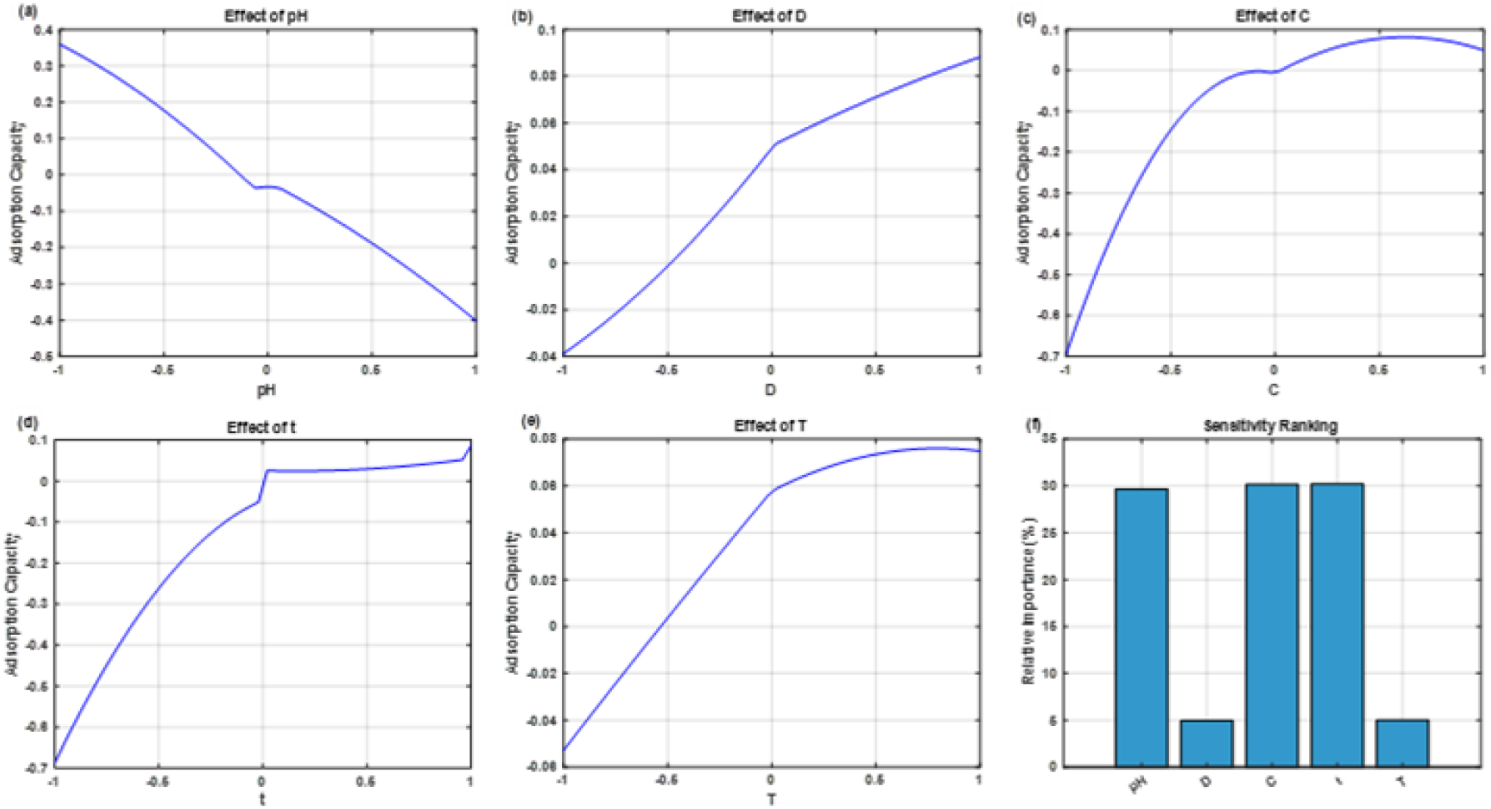
ANFIS-based global sensitivity analysis illustrating the influence of (a) pH, (b) adsorbent dosage (D), (c) initial dye concentration (C), (d) contact time (t), and (e) temperature (T) on Q_e_ for CR removal; Panel (f) presents the normalized percentage contribution of each parameter.

### 3.7 Comparative Evaluation of All Models

The predictive performances of the four data-driven models, namely MLR, ANN, ANFIS, and SVM, were compared using the violin plot in Figure 8 together with the statistical indicators in Table 3. The experimental data (green violin) show a moderately spread but centered distribution, which can be taken as the target pattern. Among the models, the ANFIS prediction band almost overlaps this experimental shape and is similarly narrow, indicating that most of its predictions lie close to the measured values and that its scatter is small. The ANN violin is only slightly wider, suggesting a comparable level of accuracy but with a marginally higher dispersion. The SVM model shows a visibly broader distribution than ANFIS/ANN, implying a few more deviations from the experimental range, but the bulk of its predictions still fall within the acceptable domain. By contrast, the MLR violin is the widest and most flattened, which reflects larger residuals and the inability of a purely linear structure to represent the nonlinear adsorption surface.

**Table 3:** Comparative Statistical Performance of All Models for Predicting CR Adsorption on SCAC

	MLR	ANN	ANFIS	SVM
R^2^	0.8667	0.9710	0.9722	0.9597
MSE	0.0314	0.0068	0.0066	0.0099
RMSE	0.1773	0.0827	0.0810	0.0995
MAE	0.1385	0.0447	0.0439	0.0682

**Figure 8:**
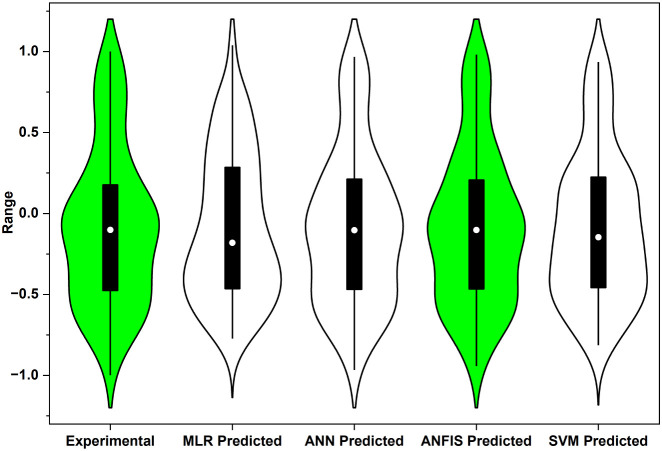
Violin plots comparing experimental and model-predicted Qe distributions for MLR, ANN, ANFIS, and SVM models in the CR–SCAC system.

These visual observations are fully supported by the numerical metrics (Table 3). ANFIS attained the highest coefficient of determination (R^2^ = 0.9722) together with the lowest error indices (MSE = 0.0066, RMSE = 0.0810, MAE = 0.0439), confirming the best pooled fit among the tested models. The ANN model produced nearly comparable results (R^2^ = 0.9710; RMSE = 0.0827), showing that a properly trained neural network model can also map the relationship effectively. The medium-Gaussian SVM, though slightly inferior to the neuro-fuzzy and ANN models, still produced a high R^2^ of 0.9597 and acceptable error levels (RMSE = 0.0995), indicating good generalization. MLR, despite its ease of interpretation, showed the smallest R^2^ value (0.8667) together with the largest RMSE (0.1773), underscoring that linear regression is inadequate for a process governed by coupled and nonlinear adsorption effects. Hence, the models can be ranked as ANFIS > ANN > SVM > MLR, showing that hybrid (ANFIS) and nonlinear ML approaches (ANN, SVM) are distinctly better suited than classical MLR for predicting CR uptake on SCAC.

## 4 Conclusion

This study compared four predictive models, such as MLR, ANFIS, ANN, and SVM, for predicting CR dye sequestration onto *Spathodea campanulata* flower–derived activated carbon using 180 batch experiments. The results clearly showed that nonlinear approaches outperformed the linear model, confirming the complex nature of dye–adsorbent interactions. Among them, the ANFIS model delivered the highest accuracy, closely followed by ANN and SVM, while MLR showed limited predictive power. ANFIS-based sensitivity analysis ranked the process variables as contact time > initial concentration > pH > temperature > dosage, highlighting that kinetic and mass-transfer effects predominantly govern CR uptake. Future research should expand data sets across multiple dyes and absorbents, couple ML models with optimization algorithms, extend predictions to fixed-bed systems, and integrate physicochemical descriptors to improve interpretability and scale-up for real wastewater treatment applications.

## Supplementary Material



## Data Availability

The data supporting the findings of this study are available at Scientific Reports (Nature) at 10.1038/s41598-025-86032-9.

## Notes

Supplementary machine learning results for CR adsorption onto SCAC; correlation plots between experimental and predicted Q_e_ values for the interaction type MLR model and medium Gaussian SVM model; RMSE values of ANFIS models with different numbers of membership functions, and statistical performance metrics of the optimized ANFIS model for training, testing, checking, and overall data sets (PDF)
