# Supplementary material for: Biomass-Derived Activated Carbon for Congo Red Dye Adsorption: Machine-Learning-Based Prediction and Comparative Evaluation
Source: ACS Omega. 2026 May 20;11(21):31650–62. doi: 10.1021/acsomega.6c02488 (PMC13234652; doi:10.1021/acsomega.6c02488)
Supplement: Supplementary file 1 [file ao6c02488_si_001.pdf]

Supplementary file

Biomass-Derived Activated Carbon for Congo Red Dye Adsorption: Machine-Learning-Based Prediction and Comparative Evaluation

Sujesh Sudarsan [a], Ramesh Vinayagam [a,*], Raja Selvaraj [a,**]

[a] Manipal Institute of Technology, Manipal Academy of Higher Education, Manipal, Karnataka - 576104, India.

*** Corresponding Authors**

* ramesh.v@manipal.edu

**raja.s@manipal.edu

List of Supplementary Figures:

Fig. S1. Correlation between experimental and predicted Q_e values for the interaction-type MLR model

Fig. S2. Correlation between experimental and predicted Q_e values for the medium Gaussian SVM model

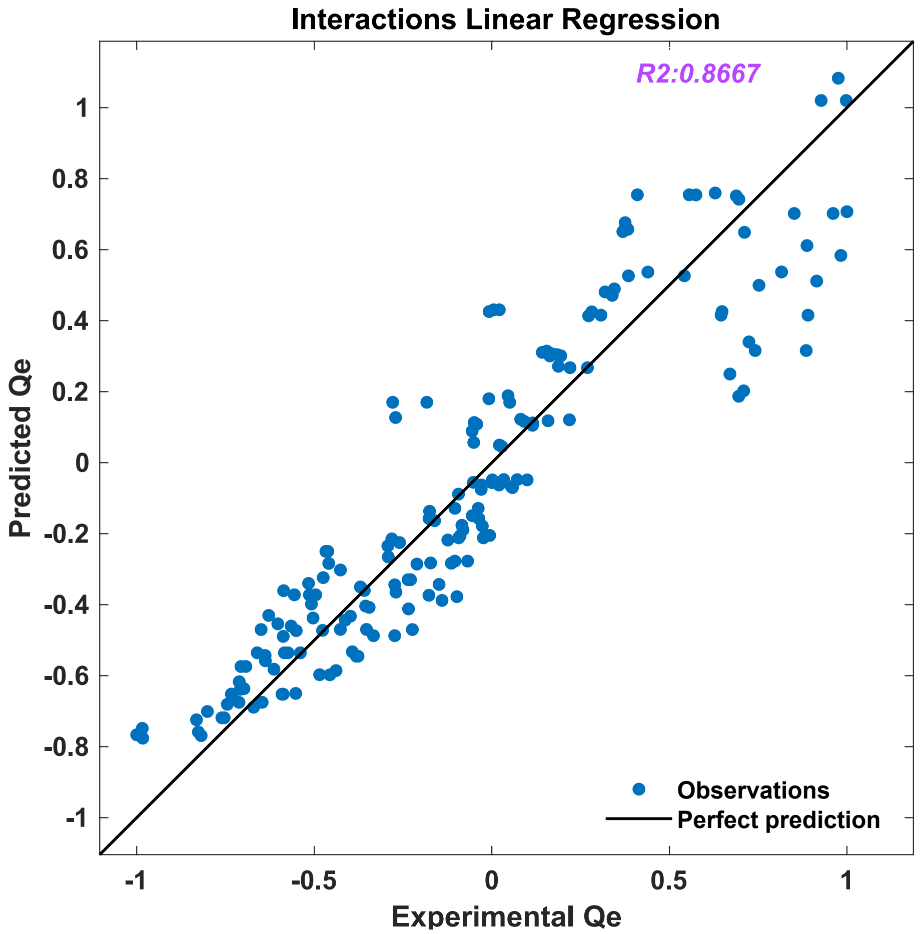

Fig. S1. Correlation between experimental and predicted Q_e values for the interaction-type MLR model

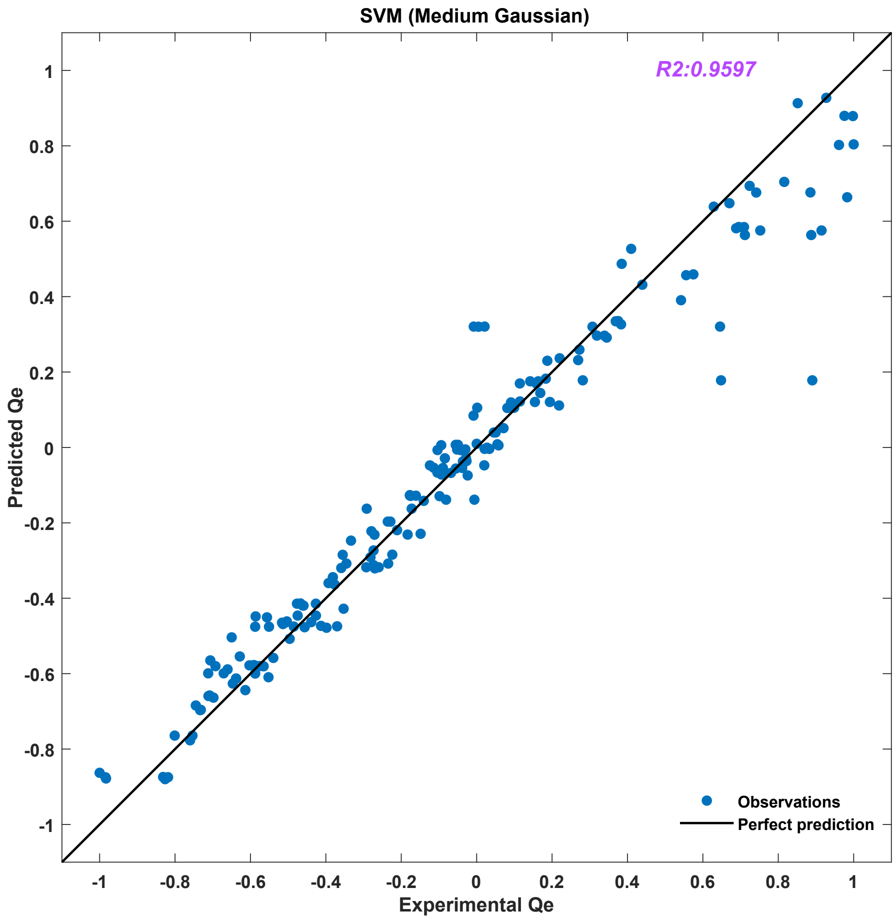

Fig. S2.

Correlation between experimental and predicted Q_e values for the medium Gaussian SVM model

List of Supplementary Tables:

Table S1. RMSE values of ANFIS (*trimf*) models with different numbers of membership functions (MFs) for training, checking, and testing datasets.

Table S2. Statistical performance of the optimized ANFIS (*trimf*, 3-3-3-3-3) model for training, testing, checking, and overall datasets.

Table S1. RMSE values of ANFIS (*trimf*) models with different numbers of membership functions (MFs) for training, checking, and testing datasets.

RMSE values			
**No. of MFs**	**Train**	**Check**	**Test**
**2 2 2 2 2**	0.1087	0.0706	0.5090
**3 3 3 3 3**	0.0573	0.0576	0.1585
**4 4 4 4 4**	0.0566	0.0597	0.1590
**5 5 5 5 5**	0.0539	0.0617	0.1583

Table S2. Statistical performance of the optimized ANFIS (*trimf*, 3-3-3-3-3) model for training, testing, checking, and overall datasets.

	**Train**	**Test**	**Check**	**Overall**
**R^2**	0.9868	0.9027	0.9857	0.9722
**R**	0.9934	0.9501	0.9928	0.9860
**MSE**	0.0033	0.0251	0.0033	0.0066
**RMSE**	0.0573	0.1585	0.0576	0.0810
